# Differences in birch tar composition are explained by adhesive function in the central European Iron Age

**DOI:** 10.1371/journal.pone.0301103

**Published:** 2024-04-03

**Authors:** Tabea J. Koch, Marion Saurel, Hervé Bocquillon, Didier F. Pisani, Lola Bonnabel, Aimée Little, Rebecca Stacey, Maxime Rageot, Martine Regert

**Affiliations:** 1 Université Côte d’Azur, CEPAM, CNRS, Nice, France; 2 YEAR Centre, PalaeoHub, Department of Archaeology, University of York, York, United Kingdom; 3 Inrap, Grand Est Nord, UMR 8546 AOROC, Châlons-en-Champagne, France; 4 Université Côte d’Azur, CNRS, LP2M, Nice, France; 5 Inrap, Midi-Méditerranée, UMR 8215 Trajectoires, Nîmes, France; 6 Department of Scientific Research, British Museum, London, United Kingdom; 7 Department of Pre- and Protohistory, Eberhard Karls University of Tübingen, Tübingen, Germany; Loyola University Chicago, UNITED STATES

## Abstract

Birch bark tar is the most widely documented adhesive in prehistoric Europe. More recent periods attest to a diversification in terms of the materials used as adhesives and their application. Some studies have shown that conifer resins and beeswax were added to produce compound adhesives. For the Iron Age, no comparative large-scale studies have been conducted to provide a wider perspective on adhesive technologies. To address this issue, we identify adhesive substances from the Iron Age in north-eastern France. We applied organic residue analysis to 65 samples from 16 archaeological sites. This included residues adhering to ceramics, from vessel surface coatings, repaired ceramics, vessel contents, and adhesive lumps. Our findings show that, even during the Iron Age in north-eastern France, birch bark tar is one of the best-preserved adhesive substances, used for at least 400 years. To a lesser extent, Pinaceae resin and beeswax were also identified. Through statistical analyses, we show that molecular composition differs in samples, correlating with adhesive function. This has implications for our understanding of birch bark tar production, processing and mode of use during the Iron Age in France and beyond.

## Introduction

Birch bark tar is the oldest known synthetic material in Europe with first finds dating to the Middle Palaeolithic [1–3], with bitumen being used in the Near-East later during the same period [4, 5]. Birch (*Betula*) bark tar (hereafter referred to as birch tar), is an organic substance with adhesive properties that can only be obtained through dry distillation of the bark from the birch tree. The oldest known examples are, or initially were, adhering to stone tools. These artefacts have fuelled much debate about the meaning of this early manufacturing technology to questions of cognitive capacity and/or cultural phenomena [3, 6, 7]. Surprisingly, evidence of adhesives chemically analysed for the European Upper and Late Palaeolithic is rare [8], a greater number of adhesive residues are reported for the Mesolithic in northern Europe [9–11]. For this latter period, birch tar is the main adhesive used in hafting technologies [11–13], and finds of tar lumps with tooth imprints raise the question of other uses such as dental care [14]. With the emergence of ceramic technologies in the Neolithic, an extension of birch tar uses takes place [15–16]. In addition to hafting [17–19], it was used to mend or seal ceramic vessels [16, 20]. Diversification can also be observed in the adhesive substances used for these purposes, with some evidence for the use of conifer resins, bitumen, and beeswax [16, 21]. The Metal Ages, and especially the Iron Age, attest to an even larger range of adhesive applications. Besides ceramic repair [22–24], adhesives were used to assemble metal objects and attach ornaments [25–28]. Birch tar in particular not only had diverse technological functions, but it also had decorative applications [29] which persisted up to the Roman period [30]. It was even still in use in early Medieval England [31]. Birch tar is therefore the oldest known human-made adhesive, fulfilling a versatile range of applications and representing the longest known continuous use of the same adhesive material throughout the archaeological record. The prevalence of birch tar as the primary adhesive in archaeological records from the Palaeolithic to the Iron Age could be attributed to its exceptional preservation compared to alternative adhesives [32]. This preference may be further enhanced by the wide availability of the raw material required given that European birches, specifically *Betula pendula* and *Betula pubescens*, are widespread species today, thriving in colder climates across northern Europe and adapting to mountainous terrains in the Pyrenees, Alps and other mountain massifs [33].

No natural exudate can be found on birch trees, and birch tar is only obtained through the distillation of birch bark at certain temperatures [9, 34, 35]. Experimental studies have explored different possibilities of how birch tar could have been made in the past, both using aceramic Stone Age methods [36–41], and methods that would use ceramic pots in the production process [34, 42, 43]. Concerning the archaeological evidence for or against either of these methods, only limited data from the Bronze Age and the Iron Age are available [44, 45]. Jakucs and Sándorné Kovács [45] report a perforated vessel covered in birch tar linked to the production of tar and more specifically to the double-pot distillation process. A similar find was discovered at the Iron Age site of Grand Aunay in France [44]. Based on molecular evidence, Rageot et al. [34] propose that the double-pot technique was already used in the Neolithic of the Mediterranean. However, the authors note that degradation and potential re-heating of tar could have an unknown influence on the chemical signatures. A recent study showed that adhesive strength can be increased through post-production re-heating of birch tar [46], but chemical alteration during such processes, as well as a way of recognising them in archaeological contexts, has not yet been assessed. Consequently, it is not only the lack of direct archaeological data on the production method, but also any post-production processing, such as reheating or mixing, that we do not fully understand yet.

Another limitation is that chemical characterisations are often undertaken in small-scale geographical contexts or on very few, often prestigious, artefacts. Relatively large-scale studies on adhesives have been conducted for the Neolithic [15, 16], but are difficult for older periods where preservation issues limit the number of samples available. For the Bronze Age only a few studies have shown the use of birch tar [22, 47, 48]; slightly more have chemically identified birch tar dating to the Iron Age [24, 25, 28, 29, 44, 49]. If period-based studies looked at adhesives on a larger scale, incorporating a diverse range of artefact types instead of being artefact-specific, they could potentially provide an even deeper insight into adhesive technologies and how they vary depending on artefact/function. Iron Age contexts provide a diverse range of applications and uses of adhesives, whilst sufficient sample sizes are available to answer some of the prevailing questions concerning adhesive technologies: are there functional advantages of using one adhesive material over another? Or are choices based on resource availability? Are different adhesives necessary for specific functions, for example, ceramic mending versus waterproofing, and how can we distinguish these differences in the archaeological record?

To address some of these questions, we conducted a large-scale study on adhesive residues serving different functional purposes in the Iron Age of north-eastern France. Our study has two main objectives. 1) We aim to analyse the chemical composition of adhesive substances used and produced in the Iron Age to broaden our understanding of the variety of substances exploited during this time. 2) We aim to explore any molecular differences that can be linked to the function of birch tar which might yield information on the production and post-production processes. To achieve this, we analysed 65 residues found on artefacts from 16 archaeological sites in north-eastern France. These sites comprise funerary and settlement contexts and date to the late Iron Age. We included samples from ceramic mending/repair, surface residues on ceramics that were interpreted as surface coating and decoration, vessel contents and adhesive lumps. CT (computed tomography) scans were undertaken on five lumps to assess the homogeneity of the material and identify any potential inclusions. Organic residue analysis including Direct Inlet–Mass Spectrometry (DI-MS) and Gas Chromatography–Mass Spectrometry (GC-MS) was carried out to chemically characterise our samples. By combining chromatographic data with statistical approaches, we aimed to shed light on the chemical differences in our sample set.

## Materials and methods

### Archaeological samples

**Fig 1:**
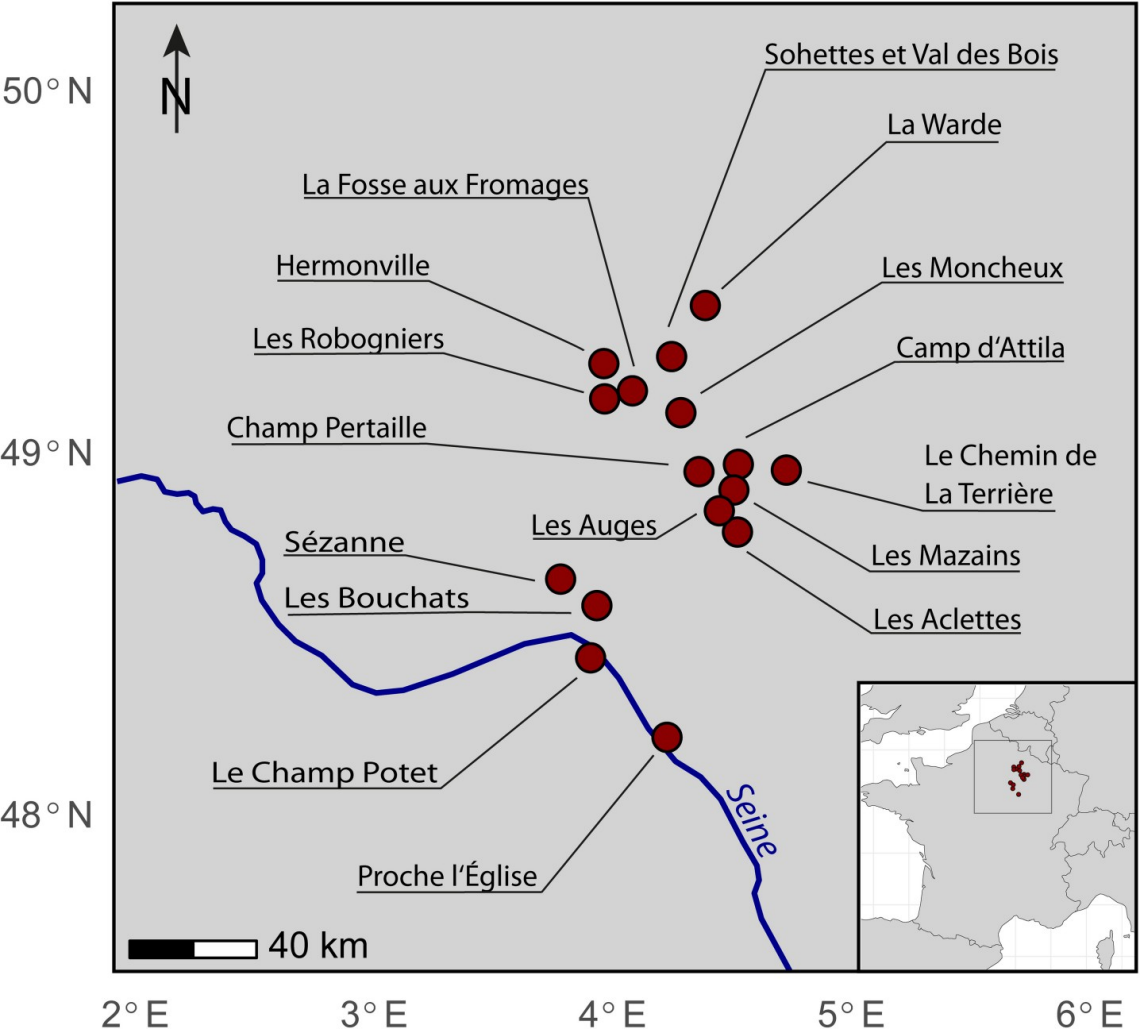
Map of France indicating the archaeological sites from which artefacts were sampled for adhesive residues for this study (Base map made with Natural Earth. Free vector map data @naturalearthdata.com).

We sampled residues from 16 archaeological sites in north-eastern France (Fig 1) for which preservation of potential adhesive residues has previously been noted (see Table 1). Nine sites are settlement contexts, and seven sites are funerary sites, predominantly dating to the late Iron Age (La Tène, 460–30 BCE), with one sample dating to the Final Bronze Age (950–800 BCE). A total of 65 objects/artefacts were selected for this study (see Table 1). The largest number of samples comes from the Oppidum Camp d’Attila [50]. To reduce any bias for statistical comparison due to sample size differences, the remaining samples originate from sites in the geographical proximity of Camp d’Attila and date to the same chronological timeframe (La Tène).

**Table 1:** List of sites/samples and relative chronology per archaeological site, with primary excavation references. Detailed information on the archaeological sites (municipality and *lieu-dits*) and samples can be found in Table 1.

Archaeological sites	Reference	Context	Chronological period	Absolute chronology (BCE)	Number of samples
ZAC de l’Ormelot	[51]	Settlement	Hallstatt B2-BC La Tène C2-D1	950–800 200–80	2
Champ Pertaille	[52]	Settlement	La Tène A-B1(a)	460–350	2
Le Chemin de La Terrière*	[53]	Funerary	La Tène A-B1(a)	460–350	1
Les Auges	[54]	Funerary	La Tène A-B1(a)	460–350	2
Les Mazains	[55]	Funerary	La Tène B	400–280	1
Les Moncheux	[56]	Funerary	La Tène A-B1(a)	460–350	2
Les Robogniers	[57]	Funerary	La Tène A-B1(a)	460–350	2
Sohettes et Val des Bois	[58]	Settlement	La Tène A-B1(a)	460–350	1
Proche l’Église	[59]	Settlement	La Tène A-B1(a)	460–350	2
Les Aclettes	[60]	Funerary	La Tène B	400–280	1
Les Bouchats	Pfister (director, ongoing excavation)	Settlement	La Tène B	400–280	1
Le Champ Potet	[61]	Funerary	La Tène B2-C1	330–200	1
La Fosse aux Fromages	[62]	Settlement	La Tène D2	80–30	1
Camp d’Attila	[50, 63, 64]	Settlement	La Tène D2	80–30	38
La Warde	[65, 66]	Settlement	La Tène C2-D1	200–80	5
Hermonville	[67]	Settlement	La Tène D1	150–80	3
Total number of samples					65

*In other publications also referred to as Le Chemin de Ternière

The selected artefacts were classified into five categories according to their adhesive function (see S4 and S5 Figs for additional illustrations of sampled objects).

The criteria used to categorise the samples into these groups are presented below.

**Fig 2:**
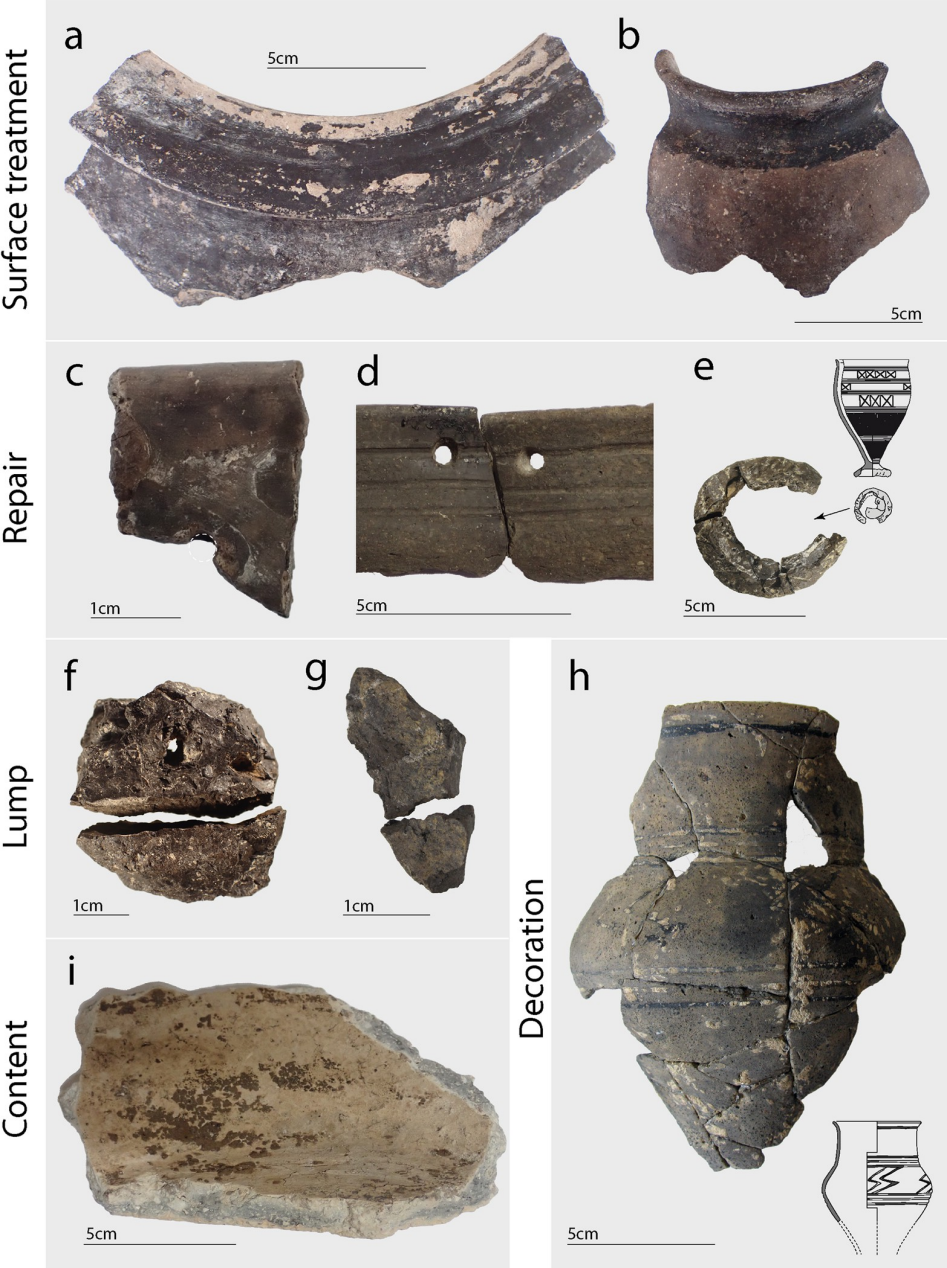
Photographs (and drawings) of some of the artefacts from which residues were sampled, as divided into function groups. a) Rim fragment with black surface treatment of a dolium from Camp d’Attila (TK8270), b) Rim fragment with black surface treatment of a vase from Camp d’Attila (TK8256), c) Rim fragment with visible adhesive residues around the ridge and a perforation hole (see white dash line) from Champ Pertaille (TK8240), d) Two rim fragments with perforation holes and black residues from La Warde (TK8229B), e) Vase foot entirely made of a dark brown matter which replaces the broken base from Les Auges (MR2600), f) Lump (broken into two pieces) with possible perforation hole from Camp d’Attila (TK8249), g) Lump from Les Robogniers (MR2606), h) Reconstructed vase fragments with black decorative pattern from Les Auges (TK8277), i) Base fragment with heterogeneous residues adhering to the interior surface from La Cheppe (TK8221). Photos by T. Koch, drawings by I. Turé and M. Saurel.

*Group 1*: *Repair (n = 16)*: These samples refer to adhesive residues that were found in the context of ceramic repair (Fig 2C–2E). This means that the residues were, for example, located on the broken edge of a sherd or filling imperfections (i. e. where fragments of the pot had chipped off). The most obvious indicators of ceramic repair are perforation holes located next to the fracture line of the sherds (Fig 2C and 2D), or the complete replacement of a broken part, such as an entire foot of a vessel, by use of adhesive (Fig 2E).

*Group 2*: *Surface treatment (n = 14)*: This group of samples corresponds to artefacts with a thin, homogenous layer of dark brown-black residue, where no distinct decorative pattern could be identified. These surface residues are mostly observed on the inside and outside around the rim of vessels (Fig 2A and 2B) and it remains unclear whether the residue served as a waterproofing agent or whether this treatment had an aesthetic function (or both).

*Group 3*: *Decoration (n = 2)*: Similar to group 2 (surface treatment), decorative residues are characterised as thin and homogenous surface residues. One sample from Les Auges (TK8277) refers to a dark substance lining incised geometrical patterns on the ceramic surface (Fig 2H). The second sample from Les Aclettes (TK8230) was taken from a black, dotted decor, although it could be related to a surface treatment. The difference to surface treatments is that a distinct decorative pattern can be identified (e. g. geometrical lines).

*Group 4*: *Content (n = 13)*: Multiple samples were taken from the interior surface of ceramic pots, where heterogeneous accumulations (or residue crusts) of residues were found on the inside of the pots (Fig 2I). Without chemical characterisation, it is unclear if these residues are food crusts/residues or potentially linked to the making or storage of adhesive substances. Three samples originate from the content of the same vase to assess potential intra-vase variability.

*Group 5*: *Lumps (n = 18)*: Lumps refer to a mass of organic substance that is not adhering to a ceramic sherd, vessel, or other artefact. These lumps vary in size, ranging from a few millimetres to up to 5 centimetres (Fig 2F and 2G).

*Undefined samples (n = 2)*: Two residue samples were taken from ceramic sherds for which no group could be allocated. One refers to a sherd that is almost entirely covered by a black organic mass that seems to have melted onto the sherd without a clear function. The second refers to a residue that dripped onto the exterior surface of a sherd, however not covering it entirely or in a homogenous manner.

### X-ray micro-computed tomography (CT)

CT scans were made on four adhesive lumps and one shaped lump used to replace a vessel foot (as shown in Fig 2F) to identify any potential inclusions within the organic materials. The scans were recorded using a SkyScan-1178 X-ray *μ*-CT system (Bruker) with a resolution of 81 *μ*m as previously described [68]. Reconstructions were produced with the NRecon 1.6.6.0 software (Bruker), using the same minimum and maximum coefficient values (0–0.055) to compare inclusion densities.

### DI-MS screening

Archaeological samples were screened for characteristic fragmentation patterns using Direct Inlet (DI)-Mass Spectrometry (MS), as proposed by Regert and Rolando [25]. For this, no sample preparation is required. A micro-grain of each sample was directly injected into the ionisation chamber of the mass spectrometer. Analyses were performed on a Shimadzu QP2010 ultra mass spectrometer. The ion source was set to 200°C. The temperature program was set to 350°C at a rate of 40°C/min, holding the temperature for 5 min. Spectra were recorded from *m/z* 50–950. The spectra were then screened for characteristic ion fragmentation patterns of birch tar, Pinaceae resin and beeswax. The presence of *m/z* 189, 424, and 408 is characteristic for triterpenoid fragmentations which may indicate a birch tar signature. The fragments at *m/z* 239 and *m/z* 285 are potential diterpenoid indicators for conifer resins and tars. Beeswax-characteristic long-chain palmitic esters can be identified through the presence of *m/z* 256 and 257, as well as *m/z* 593, 621, 639, 677, 705, and 733. If the injected sample yielded no signal, a second grain was injected to verify the result.

### Extraction and GC-MS analyses

Sample treatment and extraction protocol were based on previous studies on archaeological adhesives [16, 34]. For this, 1–3 mg of sample material was crushed and weighed. Tetratriacontane (*n*-C_34_, 10 μL of a 2 mg/mL cyclohexane solution) was added as the first internal standard. Sample powders were solvent extracted with dichloromethane (DCM) at a ratio of 2–3 mg/mL and sonicated twice for 15 min. A blank sample of DCM was added to each batch to monitor in-laboratory contamination. An aliquot of each sample was derivatised with 50 μL of N,Obis(trimethylsilyl)trifluoroacetamide (BSTFA), 10 μL of DCM and 2 μL of pyridine (heated for 60 min at 70°C). Hexadecane (*n*-C_16_, 10 μL of a 0.2 mg/mL cyclohexane solution) was added to each sample before analysis as a second internal standard. Internal standards were added to samples in which only triterpenoids from birch tar were detected by DI-MS for statistical comparison.

GC-MS analyses were performed on a Shimadzu GC 2010 PLUS gas chromatograph in splitless injection mode at a column flow of 6 mL/min. Samples identified as potential birch tar during DI-MS screening were injected into an Agilent J&W HP-5MS GC Column (30 m x 0.32 mm x 0.25 μm film thickness). The inlet temperature was set to 300°C. The oven temperature was ramped from 50°C (hold time 2 min) to 150°C at 10°C/min, and then directly increased to 320°C at 4°C/min and held for 15 min.

Samples with potential beeswax compounds (identified by DI-MS) and samples too small for screening were injected into an Agilent J&W DB-5HT GC column (15 m x 0.32 mm x 0.1 μm film thickness) using the autosampler. The inlet temperature was set to 350°C, and a temperature ramp was set to increase from 50°C (1min hold time) to 150°C at a rate of 20°C /min, then to 250°C at a rate of 10°C/min (held for 4min), and to 350°C at a rate of 20°C/min (held for 14min). No internal standards were used in these samples. The mass spectra were recorded with a Shimadzu QP2010 ultra by electron ionisation at 70 eV. The ion source temperature was set to 200°C and spectra were acquired over the range of *m/z* 50–950. Compound identification was carried out through comparison with the NIST library and data previously published [69–71].

### Statistical analyses

Samples that only contained compounds indicative of birch tar, and which had been analysed with two internal standards were further treated for statistical analysis. The total ion chromatograms (TIC) were integrated (all peaks > = 1% of the largest peak) and calibrated first to the internal standard *n*-C_34_, and then to the second internal standard *n*-C_16_. Peak integrals of identified compounds were normalised to these internal standards and served as variables for Principal Component Analysis (PCA). PCA was done on a covariance matrix. The first PCA was calculated on the chromatographic data of 33 archaeological birch tar samples from 11 sites corresponding to three of the previously defined groups: repair (n = 9), lump (n = 17) and surface treatment (n = 5). A Tukey-Kramer test was done on these samples to test the statistical significance of our results. The second PCA only included 20 samples coming from the site Camp d’Attila (lumps, n = 16; surface treatment, n = 4) to investigate whether any correlation between the function groups occurs on an intra-site level. This will at the same time allow us to assess whether differences in degradation marker abundance might be linked to the varying site contexts with different degrees of preservation. The set of variables used for our PCAs comprised 4 compounds. These compounds are allobetulin, 3-oxoallobetulane, allobetul-2-ene, and 28-oxoallobetul-2-ene. We chose to use these compounds as variables because they are not natural degradation products formed in birch bark [9] but more likely to result from the distillation process of birch tar production (directly linked to cycloisomerisation) [34]. The calibrated abundance values used for statistical analyses are listed in S2 Table. PCA and Tukey-Kramer test were performed using the statistical software JMP (Version 17).

## Results

### Homogeneity of adhesive lumps

Most of the adhesive lumps are made of a homogenous material (for sections of the CT scans see S1 Fig). The scan taken of the lump from Les Robogniers (S1C Fig) shows close to no inclusions of higher density within the lump’s matrix. The adhesive used to replace the missing ceramic foot shows the presence of inclusions with a higher density than the main matrix, with most inclusions being present on the exterior surface of the analysed fragment (S1A Fig). These denser particles might be explained by sediment adhering to the outer surface and small pieces of the ceramic possibly remaining stuck on the adhesive when it detached. Similar dense particles can be seen in S1B Fig (lump from Les Moncheux), where parts of the lump’s outer surface are denser than the inside. Both fragments from Camp d’Attila (S1D and S1E Fig) are characterised by a homogenous matrix with denser inclusions varying in size that seem randomly distributed. These lumps also show voids and cracks that might be related to taphonomy.

### DI-MS screening

Most of the 65 samples were screened by DI-MS. Twelve of them were excluded for further analysis because their mass spectrum did not yield a molecular signal indicative of the molecular constituents of interest (wax, di- and triterpenoids). Other samples were categorised as potential birch tar, Pinaceae resin or beeswax based on the presence of characteristic *m/z* ions (for birch tar: *m/z* 189, 424, and 408, for Pinaceae resin: *m/z* 239 and 285, and for beeswax: *m/z* 256 and 257, 593, 621, 639, 677, 705, and 733) and further analysed by GC-MS. An example mass spectrum, as obtained by DI-MS, can be seen in S2 Fig.

### Adhesive chemical composition

**Fig 3:**
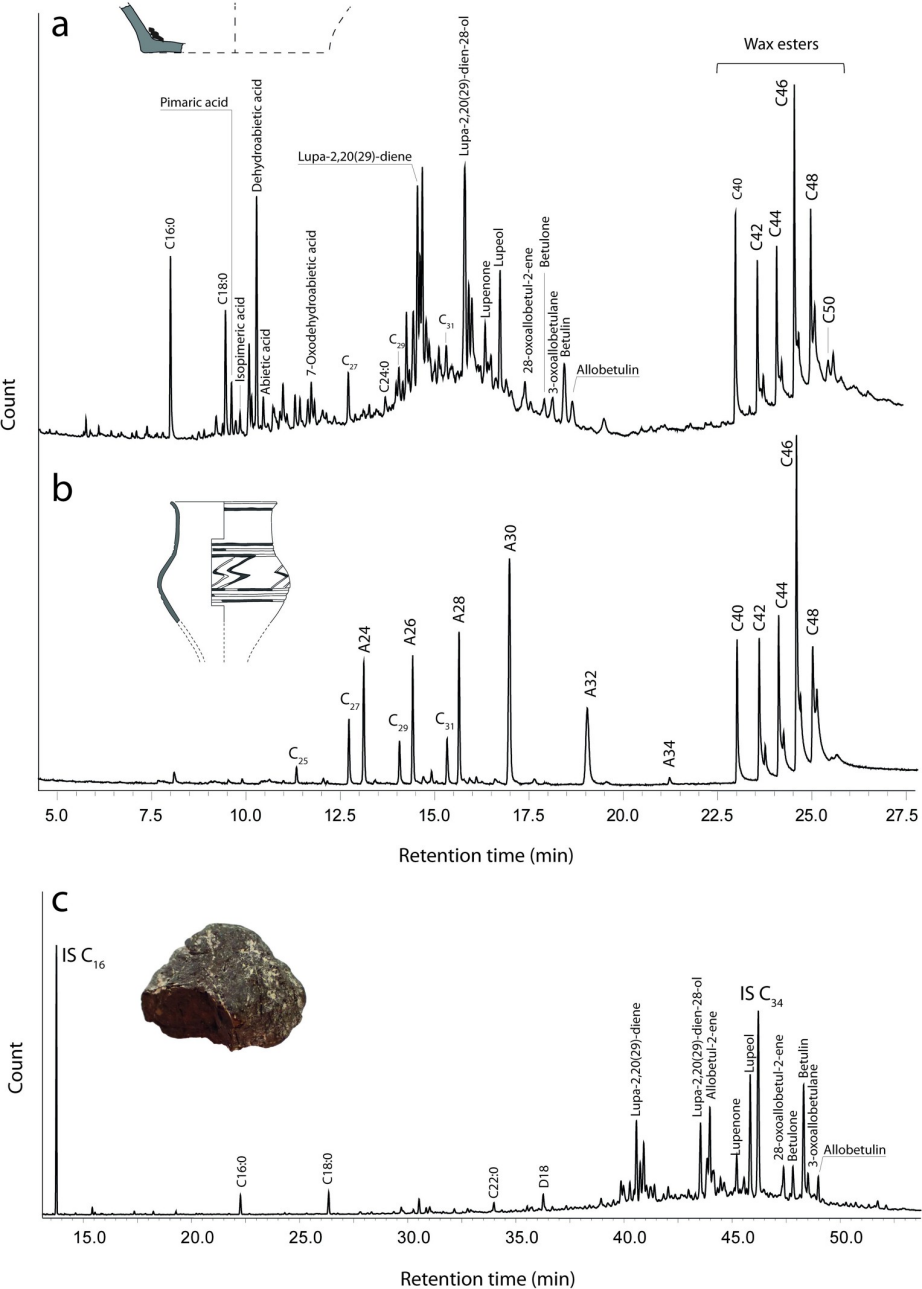
Chromatograms of adhesive residues. a) Vessel content (sample TK8221) with evidence for a complex composition typical of the presence of Pinaceae resin, birch tar and beeswax. b) Decorative pattern on a ceramic vase (sample TK8277) indicating beeswax. c) Adhesive lump only composed of birch tar (sample TK8247). DX = Diacids (X = carbon n), AX = Alcohols (X = carbon n), C_x_ = Alkanes (x = carbon n), CX:Y = Fatty acids (X = carbon n, Y = n of unsaturations), IS = Internal standard. Drawings in a-b by M. Saurel, photo in c by T. Koch.

The molecular assemblages detected by GC-MS made it possible to identify three different adhesive materials in our samples. These were present either as the sole identifiable substance or in combination with other substances (Fig 3). Forty-two samples contained compounds only characteristic for birch tar. This identification is based on the presence of specific compounds of the lupane and oleanane families, so-called bio- and degradation markers. We detected betulin and lupeol, which naturally occur in birch bark [9, 22, 72] and degradation markers that form through oxidation, dehydration and cycloisomerisation which are indicators of the transformation of birch bark into tar [9, 16, 34, 73]. Some of these degradation markers are natural degradation markers that also form by decay and are found in archaeological birch bark [9], or through dehydration and oxidation in archaeological birch tar. Some, however, are hardly formed through natural degradation and are linked to the initial production of birch tar from bark, and we regard the presence of multiple of these production-linked degradation markers as a reliable indicator to interpret the samples as birch tar (see for example Fig 3C). We identified the compounds lupa-2,20(29)-diene, lupa-2,20(29)-dien-28-ol, lupenone, betulone, which can be linked to both natural and anthropic decay; and allobetul-2-ene, 28-oxo-allobetul-2-ene, 3-oxoallobetulane and allobetulin which are more likely to result from anthropic degradation processes. We also identified a series of fatty acids (with carbon numbers between 14 and 26) and diacids (with carbon numbers between 9 and 18). Cholesterol was found in two samples, but no derivatives of cholesterol (indicative of archaeological cholesterol as opposed to post-depositional contamination) were detected. For a complete list of the detected compounds, see S1 Table.

Conifer resin, and more specifically resin from the Pinaceae family, was detected in 9 of our samples, always in combination with beeswax and/or birch tar. Pinaceae resins can be characterised through the presence of diterpenoids of the abietane and pimarane families [16, 70, 74]. We identified various combinations of the biomarkers pimaric acid, isopimaric acid, dehydroabietic acid and abietic acid, as well as their degradation markers simonellite, 7-oxodehydroabietic acid and 15-hydroxy-7-oxodehydroabietic acid (see S1 Table for detailed list of detected compounds). For 7 of the Pinaceae resin residues, we could refine the origin of the resin to the genus *Pinus*. This is based on the presence of *Pinus*-characteristic α and β seco-dehydroabietic acids and pimaric acid as a major pimarane [70]. We could not identify any abietane hydrocarbons (e. g. retene or methyl dehydroabietate) that would point towards strong heating of the resin as would be the case for wood tar [75–77].

The third substance we detected is beeswax, for which we identified specific markers in 10 of our samples. Archaeological beeswax can be identified as such by the presence of long-chain even-numbered wax esters with 40–52 carbon atoms (and the homologous hydroxy palmitates) as well as a series of *n*-alkanes with odd-numbered carbon atoms. Even-numbered fatty alcohols are also frequently reported in cases of beeswax partly hydrolysed over time or under anthropogenic action [69, 78]. Lignoceric acid (C24:0) is a long-chained fatty acid often found in archaeological beeswax [69] which we identified in 7 of the 10 samples (see S1 Table). The residue sampled from the black decorative pattern on a vessel from Sarry (Les Auges) showed that beeswax is the only component preserved in the sample (Fig 3B). The sample originating from a broken vessel handle indicates a combination of birch tar and beeswax was used to repair the ceramic (here, the tar was located on both broken edges of the handle, which would have been located on the exterior of the pot). The other associations of beeswax with birch tar and Pinaceae resin only occur in vessel contents (see for example Fig 3A).

**Fig 4:**
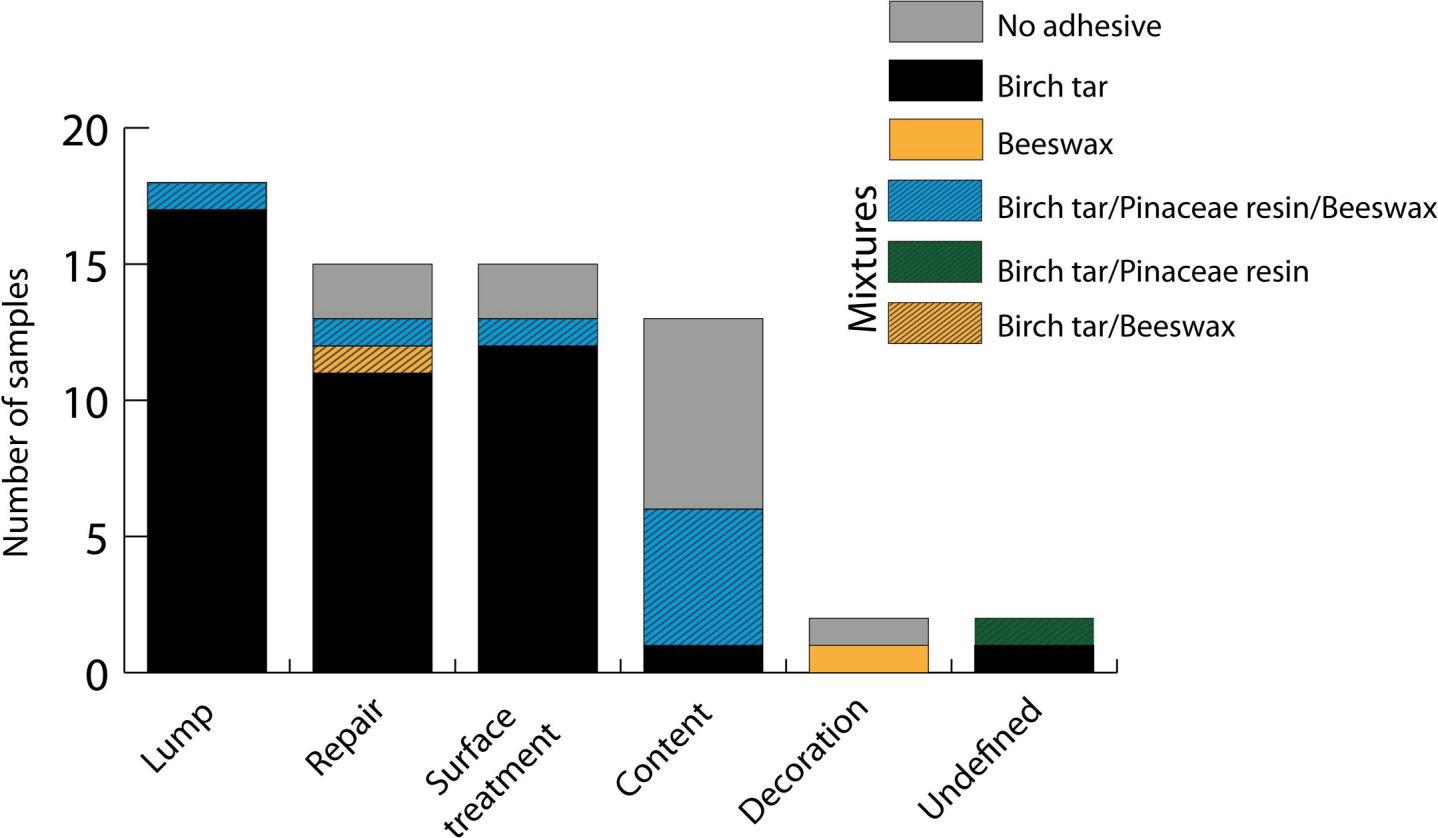
Plot of the overall results of the chemical identifications per adhesive function/group.

Fig 4 shows the distribution of the substances identified with respect to the function of the adhesives. All of the lumps could be identified as birch tar, with the exception of one lump showing a mixed chemical signature of birch tar, Pinaceae resin and beeswax. Two of the materials classified as ceramic repair adhesives yielded no result, casting doubt on their interpretation as adhesive substances. Most of the repair adhesives correspond to pure birch tar, except for the association with beeswax to repair a broken handle, and one association with beeswax and pine resin (as this sample comes from the interior, this could be linked to its contents). The residues sampled from surface coatings predominantly correspond to birch tar, with only one association of the three substances. The fewest identifications could be achieved for vessel contents. Half of the sampled residues did not yield any molecular signature. One sample corresponded to birch tar and five to a combination of birch tar, Pinaceae resin and beeswax. One sample showed the molecular signature of both birch tar and Pinaceae resin, however, the function of the adhesive on this artefact could not be clearly determined (it refers to black residues on the exterior side of a jar base).

### Statistical analyses

**Fig 5:**
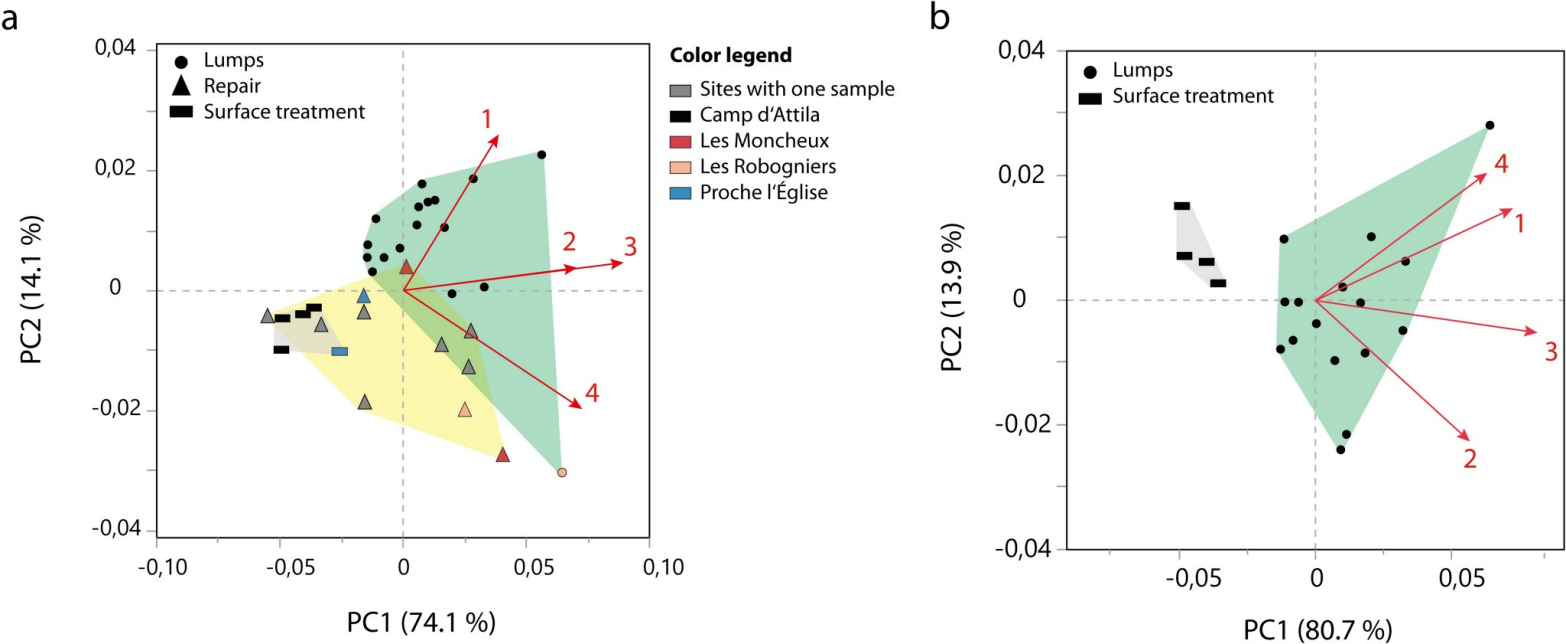
PCA plots showing the difference in adhesive function groups based on the four birch tar production markers. a) Plot of the archaeological birch tar samples from multiple sites. b) Plot of the archaeological birch tar samples from Camp d’Attila. The arrows and numbers indicate the loadings of the variables, 1 = 3-oxoallobetulane, 2 = 28-oxoallobetul-2-ene, 3 = allobetul-2-ene, 4 = allobetulin.

For samples with triterpenoid biomarkers indicative of birch tar, we conducted PCA to investigate the differences in molecular composition and abundance. If the predefined groups form distinct clusters in the PCA, it may be concluded that there are differences in molecular abundance and hence the composition of the tars. Fig 5A shows a PCA plot with three groups for which a large enough sample size was available to make statistical analysis robust: repair samples, tar from surface coating and lumps. In this plot, the lump samples form a relatively distinct cluster, although overlapping slightly with some of the repair tar samples (except for one clear outlier from Les Robogniers plotting well below the rest of the clusters; this lump was probably used to infill a defect in a ceramic, which would explain its location in the plot). The repair tar samples form a less distinct cluster, slightly overlapping with the surface tar. The surface treatment samples, the smallest sample size in this analysis, form the most distinct cluster. The overlap of lumps and repair suggests that these two groups are more similar to each other, but the lumps differ from the surface tar. As a result, Fig 5A shows that a certain difference in molecular abundance can be observed between the groups, based on samples from 11 different sites. The two samples from Les Robogniers plot together to the edge of the repair tar cluster, although they represent a repair tar and a lump (this lump is again possibly linked to ceramic repair which explains its location). Two samples from Proche l’Église plot together between the repair and surface treatment samples. Two of the repair tar samples, which originate from Les Moncheux, do not plot directly together. To investigate whether a difference can also be observed on a single site, we conducted a second PCA on samples from the oppidum site Camp d’Attila (only on lumps and surface treatment tar). The PCA score plot (Fig 5B) shows that within the same site, a difference can be observed between the two tar groups. The abundance of molecular compounds linked to birch tar production (i.e. allobetulin, 3-oxoallobetulane, allobetul-2-ene, and 28-oxoallobetul-2-ene) therefore correlates with the function of the adhesive.

The results of a Tukey-Kramer test confirm that a significant difference exists between the four production markers in relation to the function groups repair, lumps and surface treatments, as seen in Fig 5A (see Table 2).

**Table 2:** Results (*p*-values) from six Tukey-Kramer tests. Tests compare the 3 adhesive function groups (repair, lump and surface treatment) in terms of the birch tar production markers (allobetulin, 3-oxoallobetulane, allobetul-2-ene, and 28-oxoallobetul-2-ene) used to differentiate the tars in the PCA (Fig 5A).

*Allobetul-2-ene*	Lump	Surface treatment
**Lump**		0,02074
**Repair**	-0,00591	0,00667
*28-oxo-allobetul-2-ene*	**Lump**	**Surface treatment**
**Lump**		0,00769
**Repair**	-0,00308	0,00052
*3-oxoallobetulane*	**Lump**	**Surface treatment**
**Lump**		0,00483
**Repair**	0,00495	-0,01018
*Allobetulin*	**Lump**	**Surface treatment**
**Lump**		-0,00769
**Repair**	-0,00957	-0,00255

## Discussion

### Placing the data within the geographical and chronological context of birch tar use

Our results reveal that birch tar functioned as the primary adhesive within the north-eastern region of France throughout the late Iron Age. Evidence of adhesive use during the Bronze Age in this geographical context is lacking and remains sparse for the early Iron Age (Hallstatt period). Our study therefore presents some of the earliest evidence of adhesive-related technologies in this particular region. These, specifically repair adhesives, have been evidenced more frequently in burial contexts where intact vessels might favour their discovery [79–81]. During the end of the 5^th^ to the beginning of the 4^th^ century BCE, filled-in defects in pots or replaced vase parts provide some of the first indications of adhesive repair, as evidenced through our study. For example, the sample from Le Champ Pertaille (see Fig 2C) illustrates the mending technique of a double perforation to bind two pot fragments, a technique often combined with an adhesive to strengthen the repair (for details on the perforation technique, see [49]). However, an adhesive is not always identified in the context of these perforations, and the lack thereof could be explained through taphonomic factors or a binding-technology that does not necessitate an organic adhesive.

Dated to around 400–350 BCE in the Champagne region, we see the first birch tar surface treatments on mostly thin-necked vases and applied only along the exterior and interior sides of the pot’s rim. Surface treatments rarely cover the entire outer or inner side of pots (see S3 Fig). One possible hypothesis is that these pots could have been sealed with a fabric, wood or leather lid, with the adhesive functioning as a water-resistant or anti-bacterial barrier between the lid and the ceramic vessel. We are lacking archaeological evidence for this and such an adhesive function necessitates further exploratory investigation, possibly through experimental means. Two of the surface treatments, particularly those sampled on the inside of a pot, did not give a result for organic residues. This, as is the case for potential decorations, could be due to preservation issues, use of the pot as a cooking vessel, linked to the firing technique or be explained through inorganic materials used to achieve a particular visual appearance.

Although we have evidence of the use of adhesives, mostly birch tar, for repairing pots, decorations and surface treatments, and tar production is hence implied, we still do not understand how and on what scale this production took place. The evidence of birch tar in vessel contents is insufficient to ascribe their function as tar storage or production pots. Our analyses revealed considerable amounts of small birch tar lumps identified at Camp d’Attila, yet no production-related features or pots have been discovered so far. This is despite the testing of several ceramics with perforations at the base which might indicate a double-pot system and serving vases (french: *pots à déversoir*) that may have functioned as production or storage pots, neither of which revealed evidence for organic substances. This absence of molecular evidence could be linked to the suitability of our extraction method. Our protocol would not allow the identification of organic substances if these residues are carbonised/charred food residues. Interestingly, this absence of evidence for on-site production is evident across the Iron Age more generally and might be explained by off-site production of birch tar followed by transport to settlements. To assess this theory, we first need to identify possible production sites and whether molecular links can be made with tar used at surrounding sites, whether they be funerary or domestic.

When compared to the broader picture of adhesive technologies in Iron Age Europe, our results stand in line with previous studies (see S7 Fig for a geographic overview of the evidence of birch tar during the Iron Age). The practice of repairing fractured ceramic vessels has been documented since the Neolithic [16], indicating a technological continuity in Europe up to (and likely *beyond)* the Iron Age [22, 23, 82, 83] supported by our data. For the European Iron Age, evidence of tar as a surface coating on wooden vessels [84] and ceramic sherds [22, 82, 85] or as part of an ornamental pattern [29] has been relatively well-documented, which makes our identification of tar applied only to the rims a unique discovery. In addition to these uses, which are both functional and/or aesthetic, birch tar has been identified in association with metal objects ‐ specifically the ornamentation on sheaths [25, 86] and harness fittings [27, 87], and to assemble a bronze pendant [28]. Although we present no data on adhesives linked to metallurgy in this study, a future comparison of adhesive composition might yield additional insights into differences/similarities in usage between an even more diverse range of archaeological materials and object types.

### Are mixtures of birch tar with Pinaceae resin or beeswax intentional?

Besides predominantly identifying birch tar in our samples, we also identified conifer resins from the Pinaceae family and beeswax. However, the combined occurrence of birch tar, Pinaceae resin and beeswax were found almost exclusively in the form of thick heterogenous accumulations on the interior side of vessels from Camp d’Attila. Such crust-like organic preservation is rare in contexts of this region which is generally characterised by leached calcareous soils. We sampled the contents of one pot and on several sherds of this pot we identified the three substances (Pinaceae resin, birch tar and beeswax). Although it could be argued that this represents a homogeneous mixture, we cannot exclude reuse of the vessel. The presence of all three components in one lump related to vessel content could indicate an intentional mixture. The potential function of such a combined adhesive, however, remains unknown. Previous studies have found similar mixtures inside Iron Age ceramic pots (see, for example: [24, 25, 26, 44]). We support their idea that mixtures might be intentional but that the presence of multiple substances might also represent reuse throughout the pot’s life history. Intentional mixing seems more certain when identified in adhesives used for repair, in lumps, or in exterior surface treatment residues.

We only identified one mixture of birch tar and Pinaceae resin on the exterior surface of a large fragmented vessel base (TK8253A from Camp d’Attila). We analysed one sample from the interior of the same pot revealing only beeswax as an additional component of the vessel content. This might suggest that Pinaceae resin might not be related to the content but possibly represents an intentional mixture. Birch tar combined with pine resin has been reported for ceramic repair and in lumps during the Neolithic [15, 16] as well as in the Iron Age [24]. It was also found together in Iron Age adhesives related to metal work [27, 28]. Our sample was taken from a pot discovered in an area of the Camp d’Attila possibly dedicated to the manufacture of various metalwork [50, 65]. As such, future finds of adhesives on metal objects from this site could help advance knowledge concerning the intentionality of mixing birch tar and conifer resin/tar for metal-based crafts. It remains unclear whether the presence of both substances is related to resource availability; or even the production process (use of pine wood as fuel); or if conifer resins and birch tar were intentionally mixed to change/enhance the adhesive’s properties. If so, what advantages such a mixture might yield (strength, elasticity, malleability, etc.) remains to be investigated.

The presence of both birch tar and beeswax in a single adhesive repair sample from Champ Pertaille suggests that here these two materials were intentionally mixed. The broken ceramic handle displayed visible residues on the broken edges, indicating it was likely glued onto either the pot or the lid. In this case, and the handle being situated on the exterior of the pot, it is unlikely that the birch tar and beeswax were related to the contents of the vessel. Beehive products, such as beeswax, are known from other Iron Age contexts [24, 34, 88, 89], and earlier periods [90]. It is a substance previously associated with waterproofing [69, 88] or interpreted as fuel for lamps [78, 91], and mixtures with birch tar for repair, decoration and vessel coatings are known [16, 24]. Beeswax has often been referred to as a plasticising agent and experimental studies on mixtures of pine rosin and beeswax demonstrated that adhesive strength can be enhanced through mixing [92, 93]. It remains unclear if a similar effect can be observed for a mixture of birch tar with beeswax, as to date there has been no published mechanical testing for this admixture.

In the Champagne region, beeswax is hypothesised to have served as a waterproofing agent for a pot at the Warcq burial site [94]. Our finding of beeswax on its own is associated with the decoration on a vase which is unique for the local Aisne-Marne culture (c. 400 BCE) [95]. Furthermore, this finding does not align with previous analyses on geometric decorative patterns from two vessels of the slightly more recent funerary site (Warcq; La Tène C2-D1), for which birch tar (possibly in combination with conifer resin or animal fats) was used to decorate the vessel [94]. Similar results from the early Iron Age in central Italy show that birch tar was used as an adhesive to apply tin strips as décor onto ceramic pots [29]. Tin strip decorated vases are also known from the Champagne region, however the underlying adhesives have not yet been chemically characterised [96], opening up further potential for future inter-regional comparative studies. We could only identify beeswax as the main component of the residue, but we cannot exclude that, for example, ashes or charcoal might have been added for its visual appearance. Whilst we do not have any indications of ornaments attached to ceramics from our dataset, the discovery of beeswax as a decorative element adds to the application range of beeswax for a purpose that is, for the Iron Age, as far as we are aware, not previously reported.

### Does variance in birch tar composition have technological implications?

Our results show that within birch tar adhesives, a significant difference in compound abundance persists among birch tar production markers. These markers are hypothesised to form mostly during the distillation process of birch tar production [34]. If this is the case, variation in the abundance of these markers could be linked to varying production parameters. Rageot et al. [34] showed that by varying parameters in experimental set-ups (e. g. temperature, length of heat treatment, as well as quality of bark), the composition and ratios between markers also change. With our study, we tried to evaluate whether such changes in molecular composition can be linked to the function of adhesives. If a difference can be observed, it stands to reason that the production parameters may have been different, meaning different tar production techniques may have been used, or that tar may have been further processed before use. To this end, our results show that tar lumps, tar used for ceramic repair and tar used for surface treatments are significantly different from each other in terms of their production-related composition.

Lumps of birch tar, which do not show a clear previous function (e.g. hafting or repair), have been reported in a variety of archaeological contexts from the Mesolithic to the Roman Era [9, 16, 44, 47, 97–99]. These have generally been interpreted as the “primary material” obtained by birch tar production, which could then be stored, transported and used when needed. In which case the lumps from Champagne might be the material obtained through birch tar production that was then used to repair broken ceramics and applied as a surface treatment on ceramic rims, sometimes mixed with other substances such as beeswax and conifer resins for further use.

Our PCA results indicate a molecular difference between tars found in repair, surface treatments and lumps. A possible source of bias in our data could come from most samples originating from Camp d’Attila. This might play a role in the outcome of our PCA (Fig 5A), causing the difference between sample categories: samples from Camp d’Attila being lumps and surface treatments, and samples from other sites representing the repair tars. Such a difference between samples from two or more different sites could be explained through different site taphonomies or site-related production techniques. This might be supported by the two samples from Les Robogniers and Proche l’Église plotting close together, respectively (Fig 5A). However, seven repair samples originate from seven different sites, but they still plot within the repair samples cluster. In other words, they form a distinct cluster of repair samples albeit possible differences in site taphonomy or production. This means that even if site taphonomy plays a role in compositional differences, these are still smaller between the seven repair-tar sites, as opposed to their difference to the samples from Camp d’Attila. For now, with our current dataset, we can make two claims: 1) we can assess that at least for the same site, at Camp d’Attila, a difference between lumps and surface tars can be observed; and 2) that repair tars from different sites are more similar to each other than the tar categories from Camp d’Attila.

There are multiple alterations that must be considered for further interpretation of our data. A possible explanation for the difference observed between categories is that lumps represent the primary material, with some alteration to the tar taking place prior to use for repair or surface treatments. Schmidt et al. [46] show that the adhesive strength of birch tar can be enhanced through further heating/boiling which would likely be useful for repairing broken ceramics. Future chemical characterisation of such experimental data could provide a valuable asset in understanding the varying abundance of production markers. Another aspect not yet considered in great detail that could have an influence on the molecular composition of adhesives is recycling and reuse. For instance, tar employed in repairs might have been sourced from previous applications and stored as lumps for further use. Thus, it becomes plausible that some inclusions from the sediment or ceramic matrix find their way into the tar. Such a scenario appears supported by the homogeneity of tar lumps with recurring denser inclusions observed in some our CT-Scans. Reuse of tar, either from lumps or recycled from ceramics, is likely to require heating for a short duration to make it usable. However, the potential effects of low-temperature heating on the molecular composition also remain to be established.

Birch tar as a surface treatment does not necessarily require the same properties as tar for repair purposes. The surface coatings in our samples are very homogenous thin layers of residue that appear as if painted onto the ceramic matrix. We have no direct evidence of whether these pots were used for cooking and, therefore, whether repeated heating could have influenced the molecular composition. Still, the way the tar was applied does suggest a more liquid form of tar was used. Multiple authors have reported the first product of birch tar distillation with so-called *per descensum* techniques (including the double-pot technique) to result in a rather liquid and viscous tar [34, 46]. Such a consistency would be ideal to apply as a homogeneous coating onto vessels, given that the desired properties of waterproofing can be expected to be already present in this first distillate. Rageot et al. [100] suggest that in Iron Age Corsica (France), two different birch tar production techniques may have been employed: one for surface coatings and one for ceramic repair, and that they can be associated with mixtures of beeswax and pine resin/tar.

In a similar way, we argue that two hypotheses are plausible to explain our results. 1) The first hypothesis accepts that two different production techniques were employed: one to produce a liquid tar that was used for coatings of ceramic vessels ‐ either for decorative or waterproofing purposes; the other producing a more solid tar that could be stored as lumps and then further processed to be used as a repair adhesive. 2) The second hypothesis accepts that just one method of birch tar production was employed. The initial liquid tar was used to coat and decorate ceramics but was further heated to produce more solid lumps that could be stored and used for ceramic repair. This tar could be retrieved, if necessary, and stored again as lumps ready for diverse future uses.

## Conclusion

The variety of uses that birch tar had, especially in relation to ceramic repair, waterproofing and decoration, as well as its presence as lumps and fragments underscores the importance of this substance within late Iron Age society in north-eastern France. Beeswax was used for a decorative pattern and formed one intentional mixture (birch tar and beeswax) for ceramic repair, though the functional advantages of such a mixture might have remain unclear. Whilst previous findings demonstrate mixtures of birch tar and conifer resins, we have insufficient evidence that would enable confirmation of intentional mixings of these substances within our dataset. What our data does show is that birch tar is the predominant adhesive used, in keeping with previous studies from this period. Our results also help strengthen an understanding of the diversity and versatility of applications this substance had within the Iron Age material world: from ceramic mending to decoration and coating whilst also providing new insights into the *chaîne opératoire* of birch tar as an adhesive.

Analysis of chromatographic data with statistical tools allowed for the identification of differences in the molecular composition of birch tar adhesives. Such small differences were previously unknown and might indicate different production parameters based on the intended functionality. Differences in composition, we suggest, might be explained by variations in production techniques, or post-production processing steps that for now remain hypothetical. The inclusions that can be seen in some of the birch tar lumps, and especially when lumps are shaped for repair, might be indicative of the recycling and reuse of tar ‐ and it is here that significant gaps in our knowledge of this substance still need addressing. Future experiments focused on reheating, recycling, and different forms of degradation, for example, due to use and/or contextual factors will be important in enhancing our understanding of their chemical implications. It is worth pointing out that our study did not include adhesives used for assembling or decorating metal objects. Including additional artefacts, especially those made of different, non-ceramic materials, might enable an even clearer picture of how tar is used for different functional purposes. Finally, combining experimental data with chemical analyses and statistical tools is vital if we aim to address the many unknown technological and cultural aspects of birch tar adhesive production and use.

## Supporting information

S1 Fig CT sections and photos of birch tar lumps. The sample from a) Les Auges (MR2600) refers to a shaped lump used to replace a missing, broken-off, foot of a ceramic vessel. The samples from b) Les Moncheux (MR2602), c) Les Robogniers (MR2605) and e) Camp d’Attila (TK8251K) are lumps of birch tar without clear function. One lump from d) Camp d’Attila (TK8249). Photographs by T.Koch, CT Images by D. Pisani/T. Koch, drawing in a by M. Saurel.
(TIF)

S2 Fig Example mass spectrum of sample TK8259 obtained by Direct Inlet–Mass Spectrometry indicating the characteristic ions used to identify adhesive substances. (TIF)

S3 Fig Examples of pot sherds with signs of surface treatments. Samples (a) TK8255 and (d) TK8234 from Camp d’Attila with birch tar as a surface treatment on the outer and inner side of the rim, (b) sample TK8257 from Camp d’Attila demonstrating the only instance of birch tar surface treatment of the entire outer surface of a lid, (c) sample TK8244 for which no organic components could be identified. Photographs by T. Koch.
(TIF)

S4 Fig Selected illustrations of objects sampled for adhesive residue analysis dated to the earlier stages of the late Iron Age (La Tène A–B). Drawings by M. Boussel, C. Perrier, M. Saurel, I. Turé (Inrap).
(TIF)

S5 Fig Selected illustrations of objects sampled for adhesive residue analysis dated to the later stages of the late Iron Age (La Tène C2-D1). Drawings by M. Saurel and I. Turé (Inrap).
(TIF)

S6 Fig Selected illustrations of objects sampled for adhesive residue analysis dated to the latest stage of the late Iron Age (La Tène D2). Note that not all objects illustrated here yielded positive results. Drawings by M. Saurel and H. Bocquillon (Inrap).
(TIF)

S7 Fig Map of archaeological sites with published chemical identifications of birch tar. Green dots: previously published data, red dots: data from this study. List of previously published sites with details in S3 Table (Base map made with Natural Earth. Free vector map data @naturalearthdata.com).
(TIF)

S1 Table Detailed information on the archaeological sites and molecular compounds detected per sample. (XLSX)

S2 Table Calibrated abundance values used for statistical analyses. (XLSX)

S3 Table List of archaeological sites dating to the Iron Age with chemical identifications of birch tar and its function, as shown in S7 Fig. (XLSX)
