# Supplementary figures and images for: Differences in birch tar composition are explained by adhesive function in the central European Iron Age
Source: PLoS One. 2024 Apr 3;19(4):e0301103. doi: 10.1371/journal.pone.0301103 (PMC10990240; doi:10.1371/journal.pone.0301103)

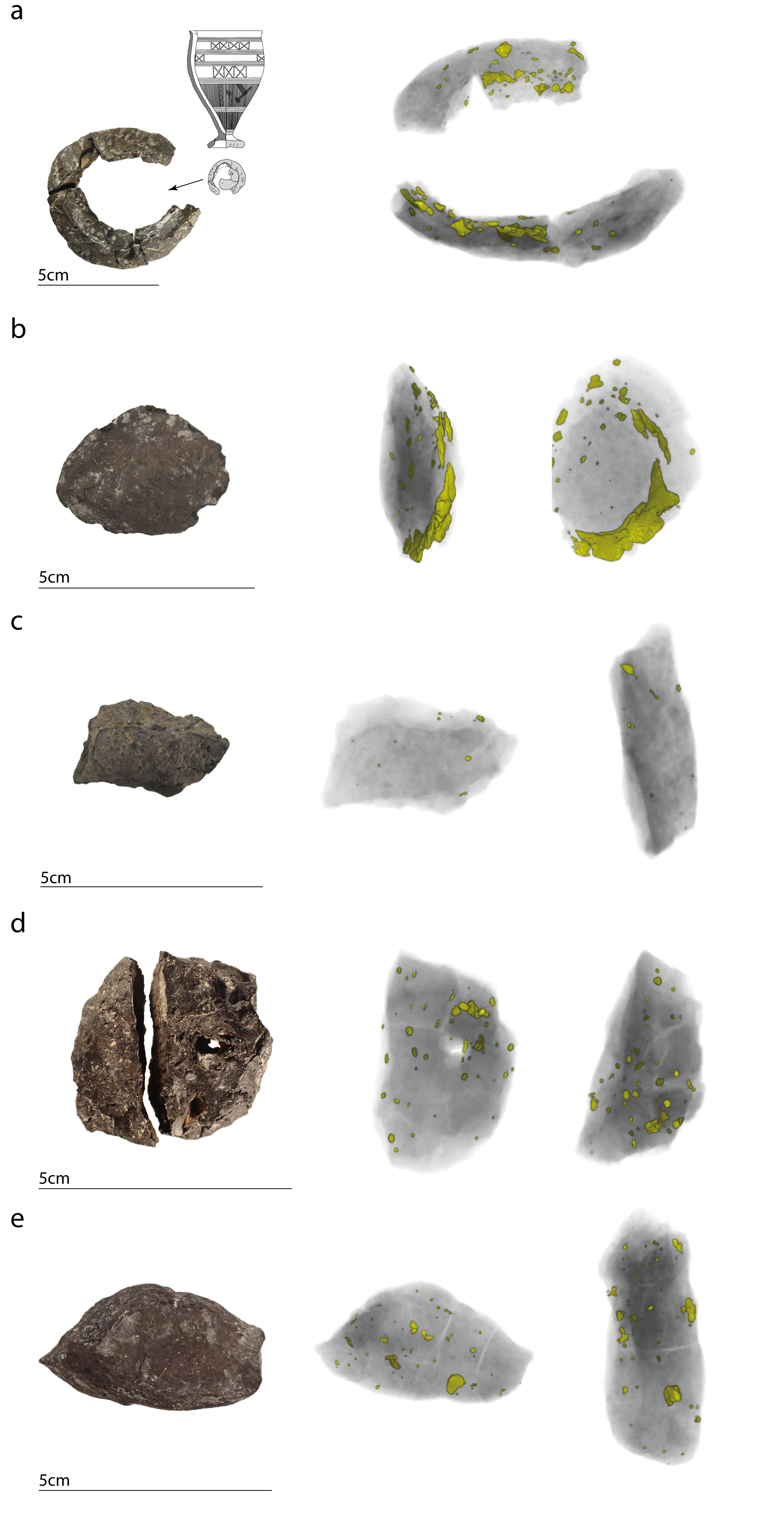

Supplement: S1 Fig — The sample from a) Les Auges (MR2600) refers to a shaped lump used to replace a missing, broken-off, foot of a ceramic vessel. The samples from b) Les Moncheux (MR2602), c) Les Robogniers (MR2605) and e) Camp d’Attila (TK8251K) are lumps of birch tar without clear function. One lump from d) Camp d’Attila (TK8249). Photographs by T.Koch, CT Images by D. Pisani/T. Koch, drawing in a by M. Saurel. (TIF) [file pone.0301103.s001.tif]

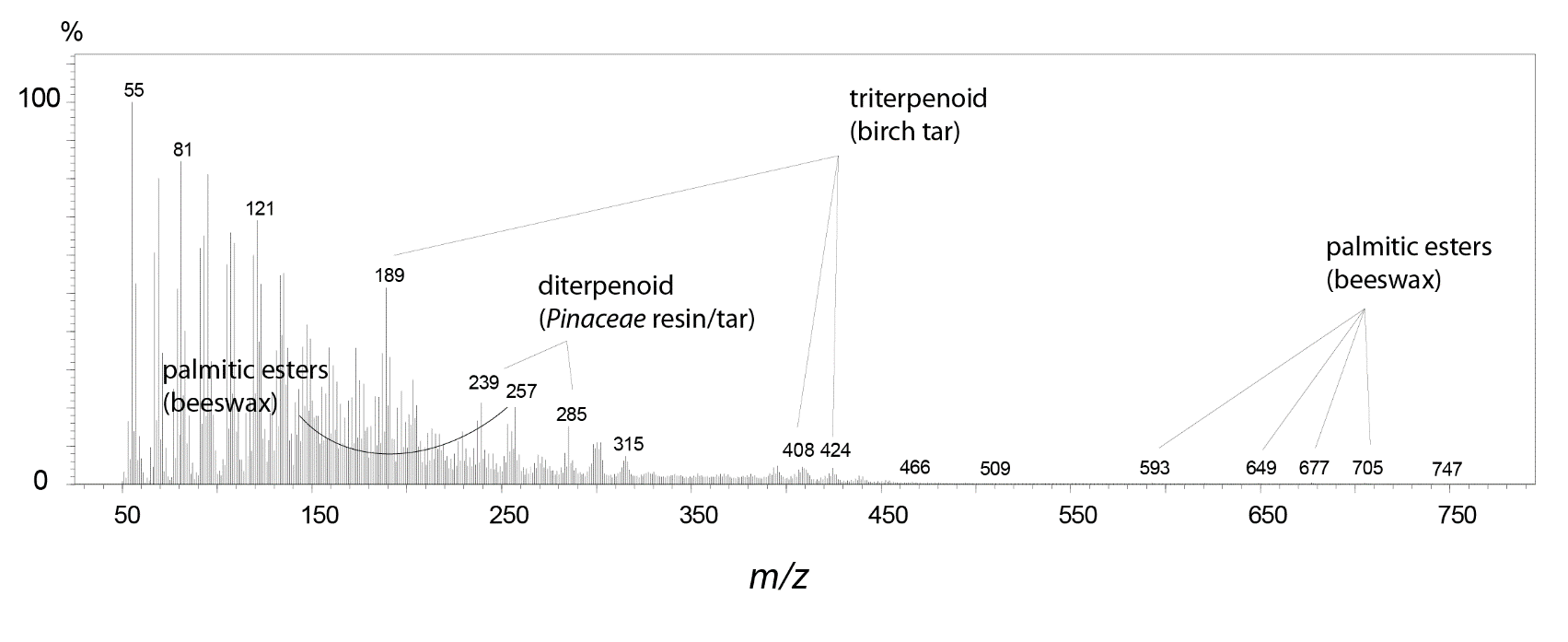

Supplement: S2 Fig — (TIF) [file pone.0301103.s002.tif]

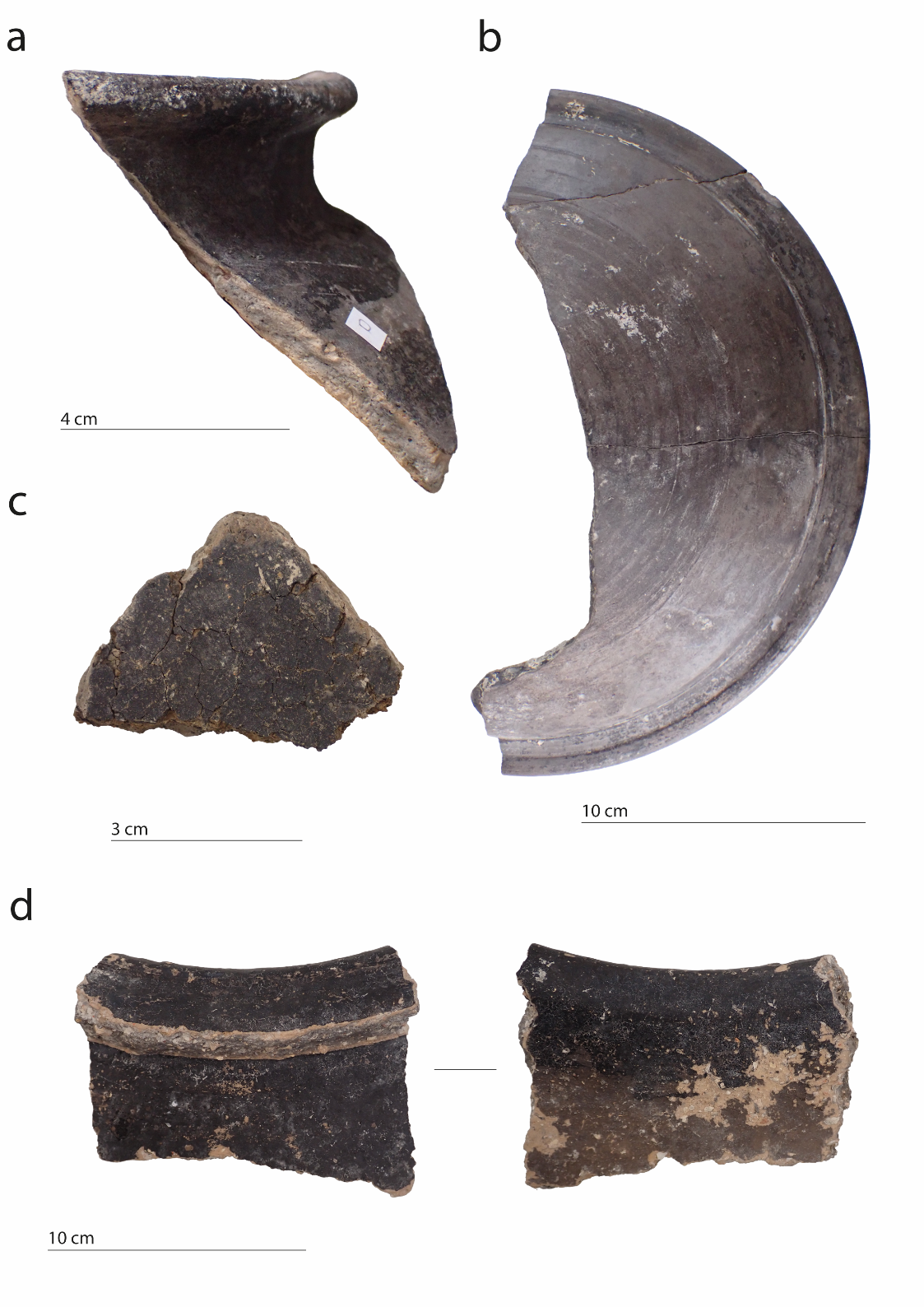

Supplement: S3 Fig — Samples (a) TK8255 and (d) TK8234 from Camp d’Attila with birch tar as a surface treatment on the outer and inner side of the rim, (b) sample TK8257 from Camp d’Attila demonstrating the only instance of birch tar surface treatment of the entire outer surface of a lid, (c) sample TK8244 for which no organic components could be identified. Photographs by T. Koch. (TIF) [file pone.0301103.s003.tif]

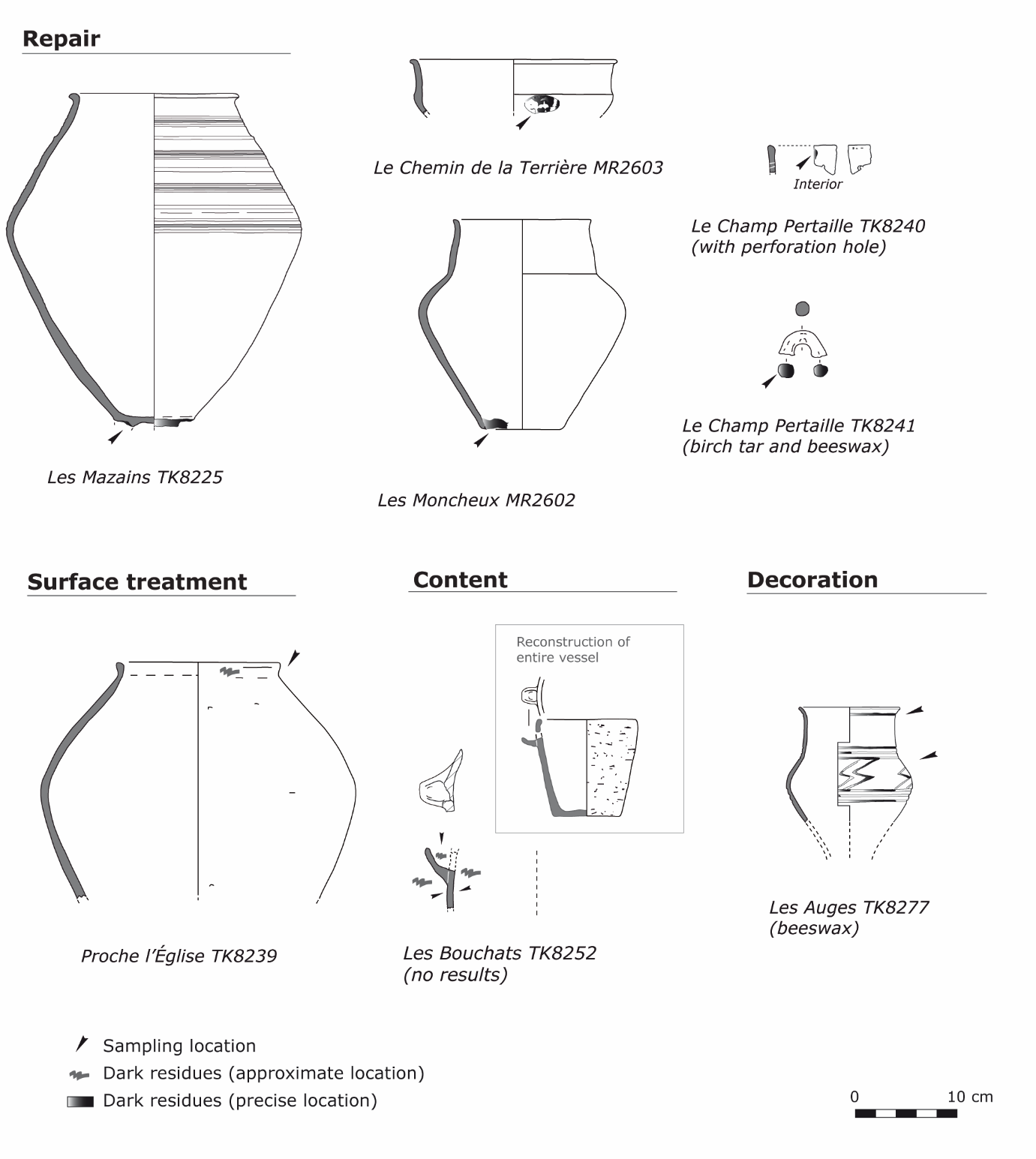

Supplement: S4 Fig — Drawings by M. Boussel, C. Perrier, M. Saurel, I. Turé (Inrap). (TIF) [file pone.0301103.s004.tif]

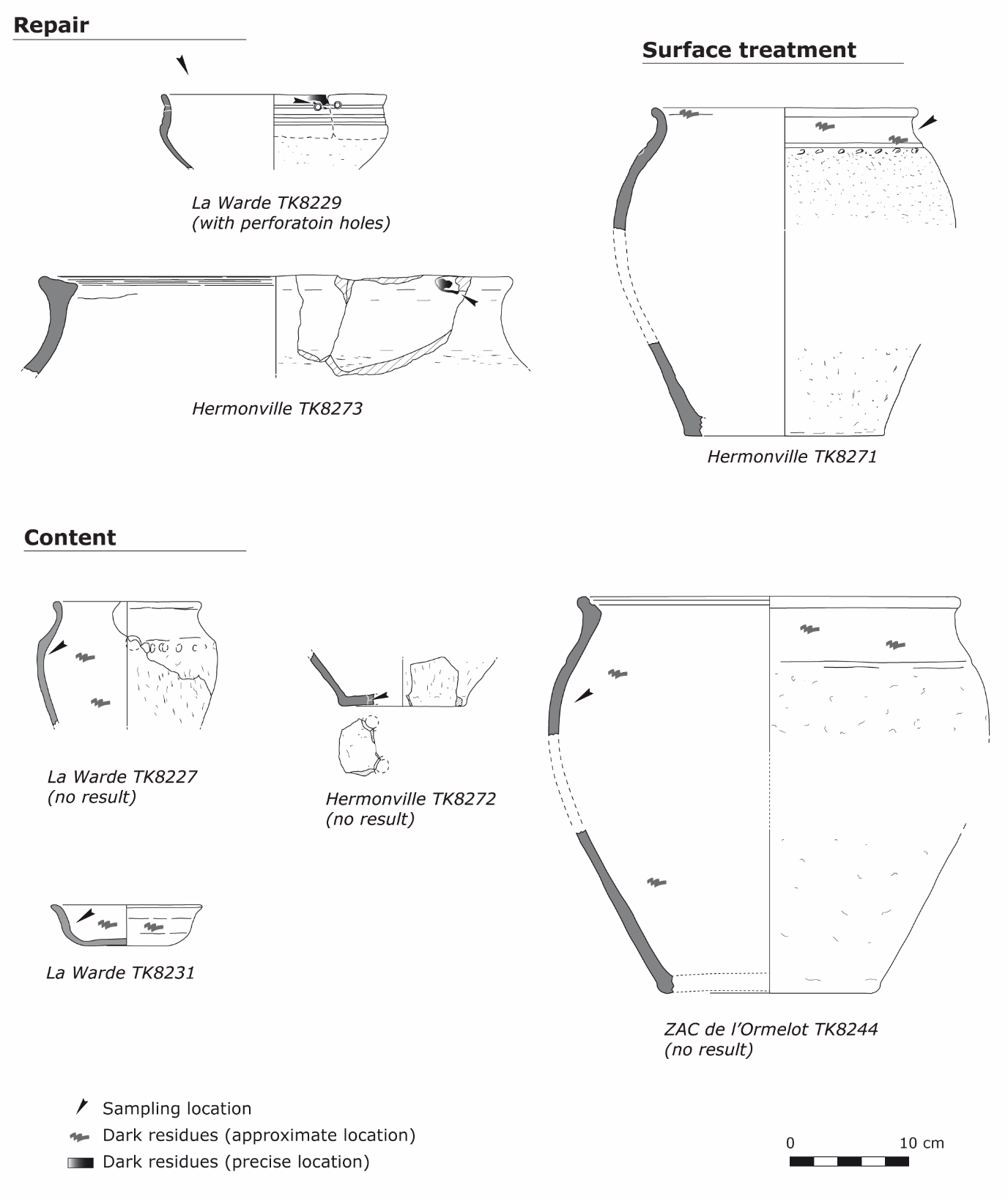

Supplement: S5 Fig — Drawings by M. Saurel and I. Turé (Inrap). (TIF) [file pone.0301103.s005.tif]

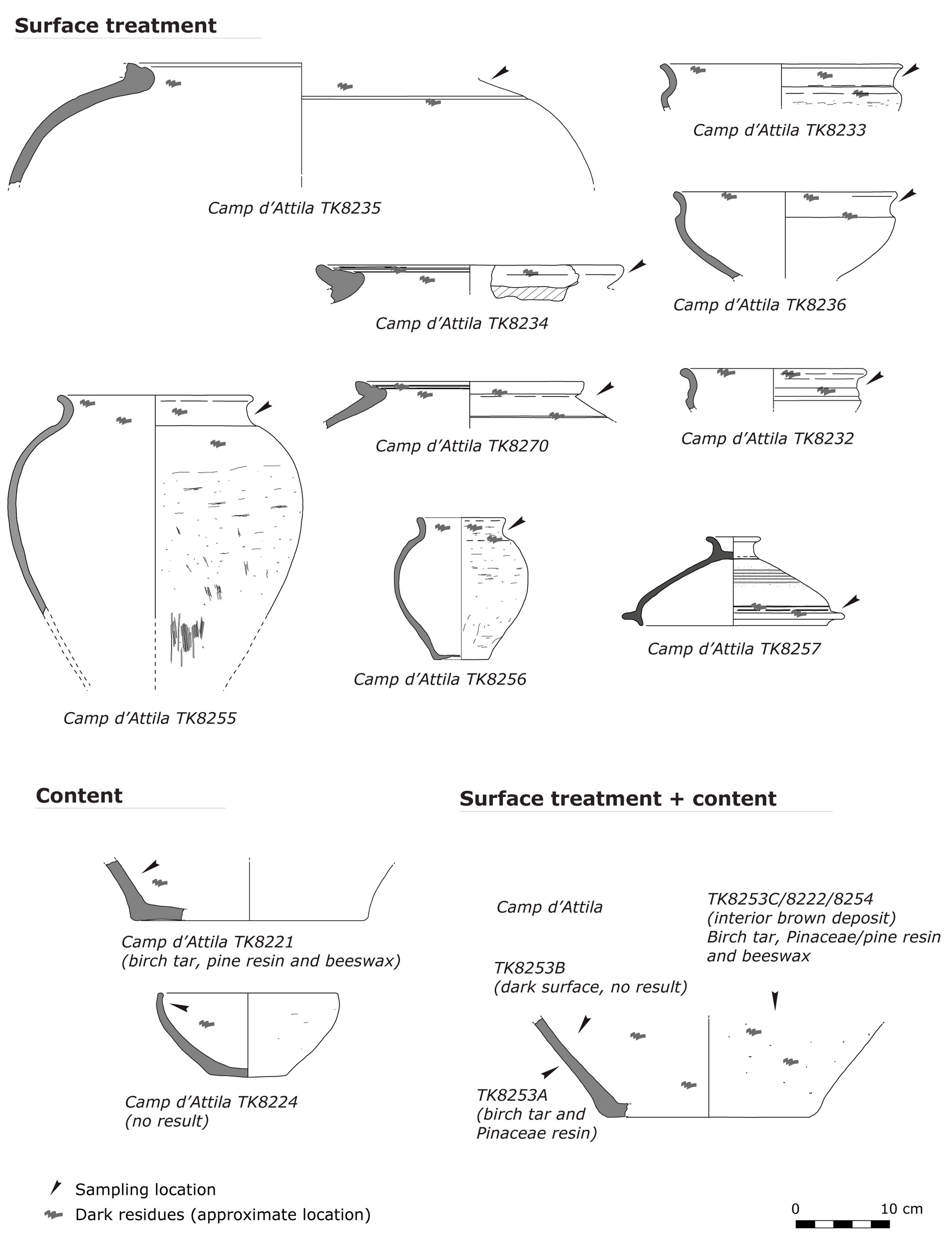

Supplement: S6 Fig — Note that not all objects illustrated here yielded positive results. Drawings by M. Saurel and H. Bocquillon (Inrap). (TIF) [file pone.0301103.s006.tif]

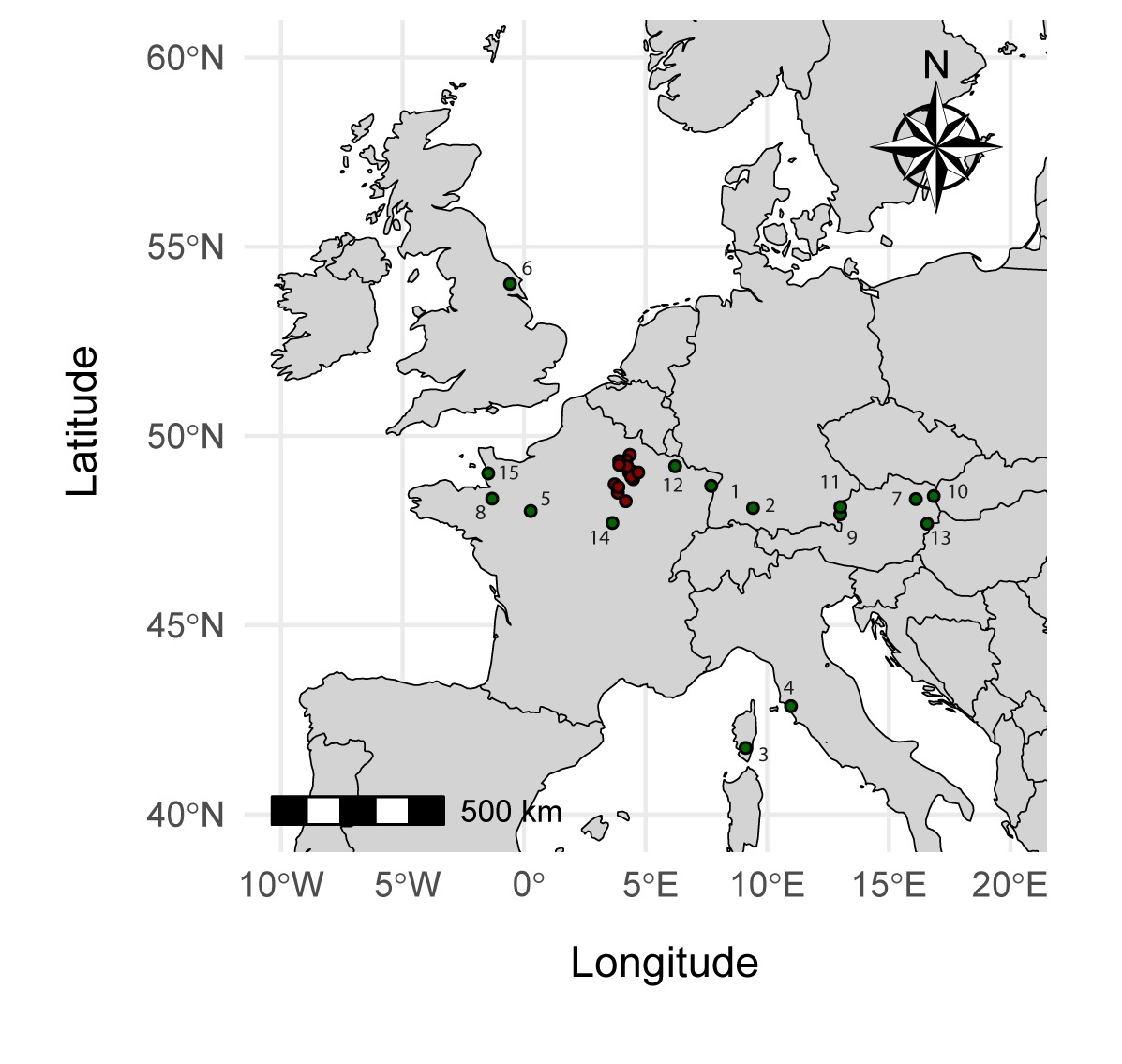

Supplement: S7 Fig — Green dots: previously published data, red dots: data from this study. List of previously published sites with details in S3 Table (Base map made with Natural Earth. Free vector map data @naturalearthdata.com). (TIF) [file pone.0301103.s007.tif]
